# Phage Lambda CIII: A Protease Inhibitor Regulating the Lysis-Lysogeny Decision

**DOI:** 10.1371/journal.pone.0000363

**Published:** 2007-04-11

**Authors:** Oren Kobiler, Assaf Rokney, Amos B. Oppenheim

**Affiliations:** Department of Molecular Genetics and Biotechnology, The Hebrew University-Hadassah Medical School, Jerusalem, Israel; Baylor College of Medicine, United States of America

## Abstract

The ATP-dependent protease FtsH (HflB) complexed with HflKC participates in post-translational control of the lysis-lysogeny decision of bacteriophage lambda by rapid degradation of lambda CII. Both phage-encoded proteins, the CII transcription activator and the CIII polypeptide, are required for efficient lysogenic response. The conserved CIII is both an inhibitor and substrate of FtsH. Here we show that the protease inhibitor CIII is present as oligomeric amphipathic α helical structures and functions as a competitive inhibitor of FtsH by preventing binding of the CII substrate. We identified single alanine substitutions in CIII that abolish its activity. We characterize a dominant negative effect of a CIII mutant. Thus, we suggest that CIII oligomrization is required for its function. Real-time analysis of CII activity demonstrates that the effect of CIII is not seen in the absence of either FtsH or HflKC. When CIII is provided ectopically, CII activity increases linearly as a function of the multiplicity of infection, suggesting that CIII enhances CII stability and the lysogenic response. FtsH function is essential for cellular viability as it regulates the balance in the synthesis of phospholipids and lipopolysaccharides. Genetic experiments confirmed that the CIII bacteriostatic effects are due to inhibition of FtsH. Thus, the early presence of CIII following infection stimulates the lysogenic response, while its degradation at later times ensures the reactivation of FtsH allowing the growth of the established lysogenic cell.

## Introduction

Proteolysis of key regulatory factors is an important control element of gene activity both in eukaryotic and prokaryotic cells. In bacteria degradation by ATP-dependent proteases, belonging to the AAA+ superfamily, participates in regulation of many developmental pathways: the heat shock response, starvation adaptation, DNA damage repair, capsular polysaccharide biosynthesis, sporulation and control of bacteriophage development [1]–[5]. Specific adaptor proteins are known to modify the interaction of substrates with ATP-dependent proteases (reviewed in [6]–[8]). However, there are only three known intracellular inhibitory polypeptides. The phage T4 PinA protein inhibits the Lon protease [9], and both the *Bacillus* species sporulation regulator SpoVM [10] and the phage λ CIII [11], [12] inhibit the FtsH protease. Both FtsH inhibitors, SpoVM and CIII, were predicted to form amphipathic α helices and are degraded by FtsH [12]–[14].

The FtsH protease is the only essential ATP-dependent protease in *E. coli*. [15], [16]. It is a membrane-bound homohexamer enzyme made of three major domains: a trans-membrane domain, an ATPase domain and a protease domain. FtsH is complexed with HflKC forming an FtsH_6_-HflKC_6_ holoenzyme, which is present in the cell in less than 100 copies. FtsH degrades membrane proteins [17] and a number of cytoplasmic proteins such as LpxC, σ32, SsrA-tagged proteins and the bacteriophage λCII and CIII proteins. Degradation of LpxC by FtsH is required for *Escherichia coli* viability, as the levels of LpxC are essential for maintaining the balance in the synthesis of phospholipids and lipopolysaccarides.

Bacteriophage λ infection may activate either the lytic or the lysogenic developmental pathway [18], [19]. In λ infection, physiological conditions as low temperature [20], starvation of the cells and high multiplicity of infection (MOI) [21] are known to favor lysogeny. A few phage functions are specifically required for the lysogenic response [22], [23]. The CII transcriptional activator, which is a key regulator of the lysis-lysogeny decision, induces three promoters essential for the lysogenic pathway. CII is required for the initial synthesis of the CI repressor from the pE promoter and of the integration protein Int, from the pI promoter. In addition, CII activates the paQ promoter and thus inhibits the Q antiterminator essential for lytic gene expression. The CII transcriptional activator is subjected to multilevel controls [19]. High levels of the CII protein, that are required for the activation of the lysogenic developmental pathway, are facilitated by λ CIII, a 54-residue peptide which protects CII from rapid degradation by FtsH [11], [12]. The CIII protein was also shown to induce the heat shock response by stabilizing σ32 [13]. A 24-amino acid (residues 14–37) region of the λ CIII protein, which is essential and sufficient for CIII activity, was predicted to form a conserved amphipathic α helix [13]. *In vitro* assays in a purified system showed that CIII inhibits FtsH proteolysis activity and can be degraded by the enzyme [12].

In this work we present novel findings on the structure and mechanism of action of CIII *in vitro* and analyze its *in vivo* functions. We demonstrate that CIII possesses an amphipathic alpha helical structure. It is present in solution as higher order complex structures and acts as a competitive inhibitor of FtsH by preventing the binding of CII. We further show that both FtsH and HlfKC contribute to the down-regulation of CII activity following infection. Moreover, real-time measurements of GFP reporter fusions demonstrate that CIII levels have a profound influence on CII stability *in vivo* suggesting that CIII may control the lysis-lysogeny decision. Finally, we demonstrate that the cause for the bacteriostatic effect of CIII is inhibition of FtsH that affects the balance in lipid membrane composition.

## Results

### Conservation of CIII

**Figure 1:**
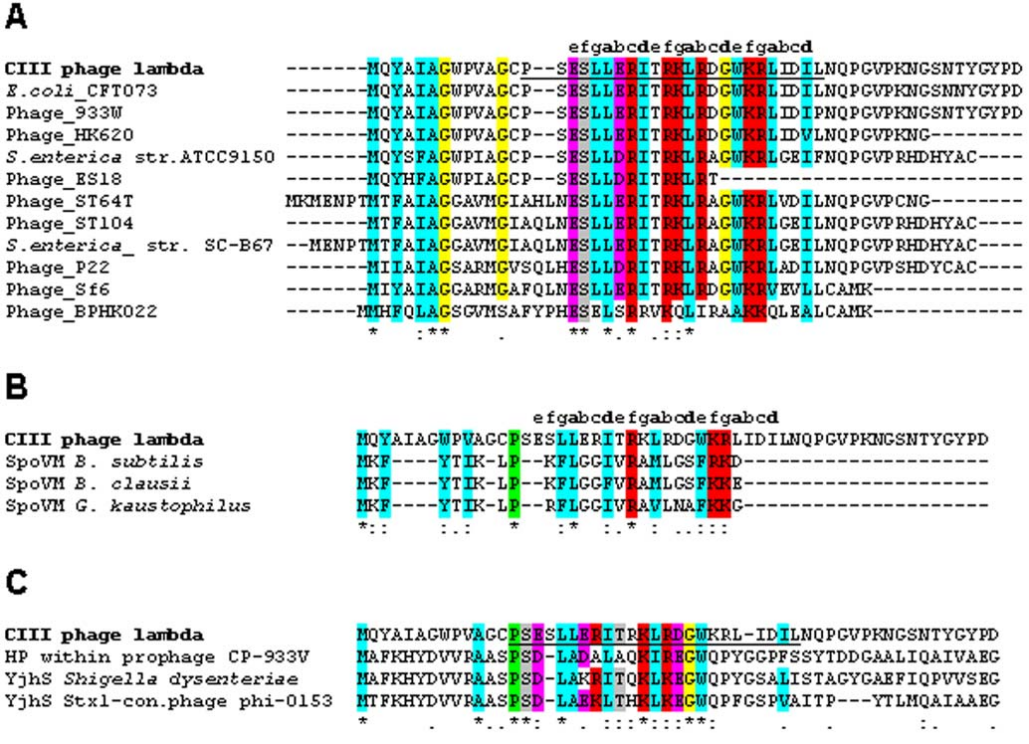
Conservation of CIII in phage and bacteria. The multi sequences alignment was done using the ClustalX (version 1.81) program. Conserved or partially conserved residues are colored according to their biochemical properties. The essential region for CIII activity is underlined and the positions predicted coiled coil (by the MARCOIL) is marked above the sequence. (A) Alignment of CIII proteins from different phages and prophages (represented by the phage name or the bacterial strain carrying the prophage, respectively). CIII proteins were collected from the BLAST program by using the lambda CIII sequence as a query for the non-redundant database. A separate search using the HK022 CIII sequence was done. The HK022 CIII sequence is identical to that of HK097A and H-19B. A complete list of all the phages and prophages in which CIII homologs were recognized, is presented in the supplementary material. (B) Alignment of CIII and SpoVM proteins was carried out with the lambda CIII protein aligned with three SpoVM proteins from different Bacillus strains. (C) Alignment of CIII and YjhS hypothetical proteins that carry the DUF1737 and DUF303, was carried out. The three YjhS-like proteins were derived from different Shigella toxin carrying phages. The HP stands for hypothetical protein without a specific name.

The recent surge in sequencing of bacteriophages and of prophages yielded an increasing number of conserved *cIII* encoding genes. These can be classified into two classes: the λ CIII protein homologs and the HK022 CIII homologs (Figure 1A). The larger λ CIII group includes the phage 933W, which is the best studied phage encoding for the Stx2 Shiga toxin (see [24]). The HK022 CIII [25] group includes in addition, only HK097 and H-19B that encode for the Stx1 Shiga toxin. In figure 1A only the HK022 CIII is shown as all three proteins have identical sequence. The 24-amino acid (residues 14–37 underlined in figure 1) region essential for λ CIII activity [13] is the most conserved sequence. This sequence was previously predicted to form an amphipathic α helix containing a short coiled coil structure [13]. In this region the a and d coiled coil positions are mostly occupied by hydrophobic amino acids. Additionally, a relatively high number of conserved positive and negative residues are found. Interestingly, five spaced residues, including two glycines, are highly conserved at the amino terminus. The function of these residues is not known.

The other FtsH inhibitors, the conserved SpoVM from *Bacillus* species, are also predicted to form an amphipathic α helix. Figure 1B shows alignment of CIII with the SpoVM peptides. The alignment suggests that these inhibitors are structurally similar.

Search of the genome database for CIII-related peptides, using the region essential for CIII activity as query, yielded one protein domain of unknown function, DUF1737 (Figure 1C). This domain was found at the N-terminus of 53 bacterial and bacteriophages open reading frames, and is joined to a domain of unknown function, DUF303, in 42 cases. These open reading frames are found downstream to Shiga toxin encoding genes in Stx converting phages, indicating a possible role in bacterial virulence.

### Purified CIII possesses an α helical structure and is able to oligomerize

**Figure 2:**
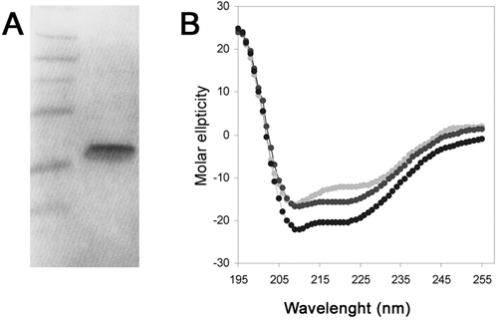
Circular dichroism of CIII. (A) 200 pmol of purified His_6_-CIII (right lane) were loaded on 4–12% NuPAGE (invitrogen) and visualized by Coomassie staining. On the left lane protein size marker was loaded (sizes 49, 38, 28, 14, 6 and 3 kDa). (B) The Circular dichroism spectrum (wavelengths 195–255 nm) of the purified His_6_-CIII at 3.5 µM (light gray), 27 µM (dark gray) and 40 µM (black).

**Figure 3:**
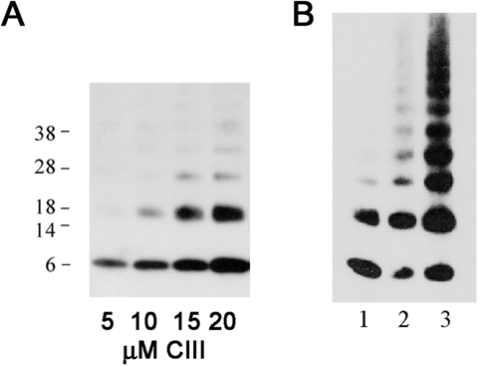
Oligomerization of CIII. (A) 10 µl of different concentrations of purified His_6_-CIII protein were loaded on 4–12% NuPAGE (invitrogen) and visualized by western blotting using commercial antibodies against the His6 tag. (B) 10 µM of His_6_-CIII was incubated in the presence (lanes 2,3) or absence (lane 1) of 0.25% Glutaraldehyde and the cross-linking reaction was stopped after 1 or 5 minutes by 200 mM Glycine (lanes 2 and 3 respectively). The reactions were loaded on 4–12% NuPAGE (invitrogen) and visualized by western blotting using commercial antibodies against the His6 tag.

A highly purified preparation of His_6_-CIII was prepared as previously described [12] (Figure 2A). The CIII structure was analyzed in solution by circular dichroism (CD) at 37°C (Figure 2B). Analysis of the CD spectra suggests that the protein forms an α helical structure. Furthermore the α helical content increases from 56% to 78% as the concentrations of CIII increases from 3.5 µM to 40 µM, suggesting that interactions between CIII protomers contribute to secondary structure stability. Figure 3A shows that multiple oligomers can be visualized using western blot analysis and that CIII oligomers can partially resist electrophoresis in the presence of SDS. To determine the oligomerization state of CIII in solution, His_6_-CIII was chemically crosslinked by glutaraldehyde, the oligomers separated by electrophoresis and visualized by His_6_ antibodies (Figure 3B). A ladder of ordered protein bands indicating increasing numbers of monomers up to a 12mer could be clearly detected. We conclude that CIII is present in solution in multiple oligomeric states. Furthermore, we detected CIII oligomers by electrophoresis of cells following λ infection (data not shown).

### CIII functions as a competitive inhibitor of FtsH

**Figure 4:**
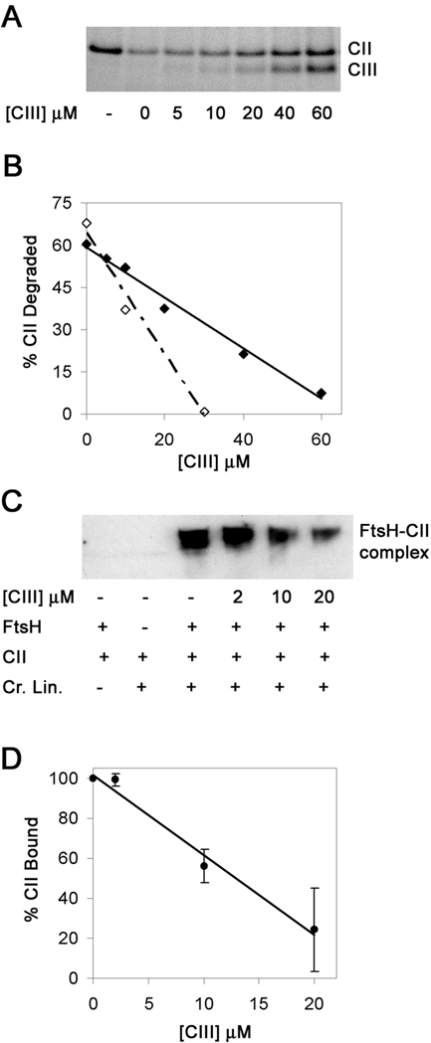
Purified CIII acts as a competitive inhibitor of FtsH. (A) 4 µM of CII were incubated with 1 µM GST-FtsH at 42^0^C for 5 minutes in the presence of increasing concentrations (as specified) of His_6_-CIII. The reactions were loaded on 4–12% NuPAGE (invitrogen) and visualized by Coomassie stain. First lane indicates the amount of CII at starting time. (B) The CII levels remaining after degradation experiment shown in A, were calculated using the “NIH image” program and plotted versus CIII levels (full diamonds and full lines). A similar experiment with only 1 µM of CII being degraded is shown in empty diamonds and a dotted line. (C) The binding assays were done in the presence of 10mM AMP-PNP as described in the materials and methods. The reactions were loaded on 4–12% NuPAGE (invitrogen) and visualized by western blotting using antibodies raised against the CII protein. (D) CII levels bound in complex to GST-FtsH were calculated using the “NIH image” program and average from two different experiments was plotted versus CIII levels. The error bars are +/− SD.

To determine the mechanism of FtsH inhibition by CIII we performed an *in vitro* proteolysis assay of CII in the presence of increasing CIII to FtsH ratios (Figure 4A). We found a linear inverse correlation between CIII concentration and the ability of FtsH to degrade CII. These results suggest a simple dose-dependent inhibition. We found that degradation of 1 µM CII is completely inhibited by 30 µM CIII whereas for 4 µM CII, more than 60 µM CIII are required for complete inhibition (Figure 4B). These findings suggest a competitive inhibitory mechanism. To test if CIII competes with CII at the level of binding to FtsH we carried out cross-linking experiments. We found that CIII inhibits the formation of CII-FtsH complexes (Figure 4C and D). The concentrations of CIII required for inhibition of CII binding to FtsH correlate with the levels needed to inhibit CII proteolysis, suggesting that CIII acts by competing for the substrate recognition site of FtsH. During the proteolysis assay the CIII concentrations also decrease, therefore when comparing the proteolysis assay to the binding assay higher levels of CIII are required in the former to obtain similar inhibition.

### The *ftsH* and *hflKC* mutant strains allow over expression of CII independent of CIII

**Figure 5:**
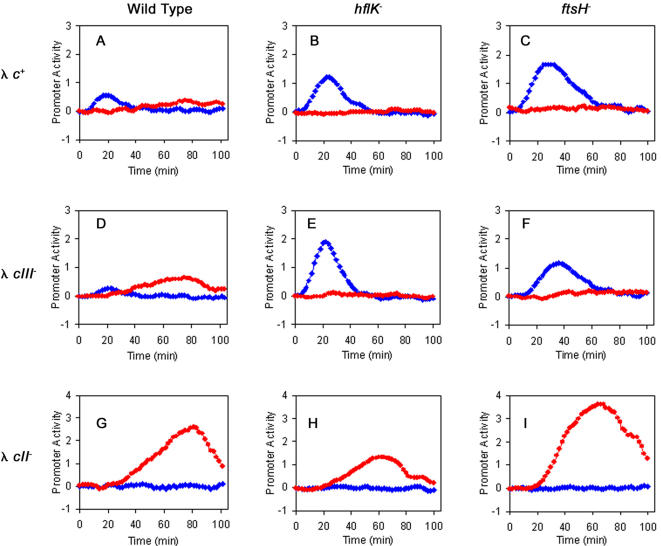
FtsH and HflA affect the λ genetic network. CII activity is reported by pE-*gfp* fusions (blue diamonds), whereas Q activity is reported by pR‘-tR‘-*gfp* fusions (red circles). The promoter activity as function of time of cultures of wild type strain (A, D, G) *hflA*- strain (B, E, H) or *ftsH* deleted strain (C, F, I) infected with λ*c+* (A, B, C), λ*cIII^−^* (D, E, F) and λ*cII^−^* (G, H, I) is shown. All experiments were carried out as described in the materials and methods at a MOI of 6.

Following phage infection, CII and CIII are required for efficient lysogeny. CII induces the expression of the CI repressor from the pE promoter. FtsH, which is found in holoenzyme complexes with HflKC, degrades both CII and CIII. It was shown that mutations in *ftsH* or *hflKC* promote high frequency of lysogenization that is independent of CIII, while mutations in the *cII* gene allow for phage growth [26], [27]. To obtain quantitative data on the role of CIII in the absence of FtsH or HflK we carried out infections of bacterial mutant strains with λ wt and mutants under conditions that greatly favor the lysogenic pathway. CII activity was assayed by a real-time kinetic readout from the pE-GFP reporter (Figure 5, blue lines) [28]. In parallel we assayed the lytic pathway by measuring Q activity using the pR′-tR′-GFP reporter (Figure 5, red lines) [28]. The results show that levels of CII in both *ftsH^−^* or *hflKC^−^* strains are much higher than those obtained following infection of the isogenic wild-type host. Furthermore, both host mutants show efficient suppression of the *cIII* mutation, suggesting that in the absence of FtsH and HflKC, CIII is dispensable. However, the loss of CII activity, following a peak, suggests that additional proteases participate in the degradation of CII.

We have previously shown that the Q function, measured from the pR′-tR′-GFP, is very low under conditions favoring the lysogenic pathway, mainly due to the presence of high CII levels [28]. Consistent with our previous findings, Figure 5 shows that infections with a λ *cII^−^* mutant phage lead to high Q activity. Q activity is observed even in the absence of FtsH or HflKC, suggesting that the effect of these proteins on the lytic pathway is mediated mainly by CII. We note however, that infections of the *hflK* mutant result in significantly lower levels of Q activity. Presently, we do not have a simple explanation for this result.

### The level of CIII activity controls the output from the CII-dependent pE promoter

**Figure 6:**
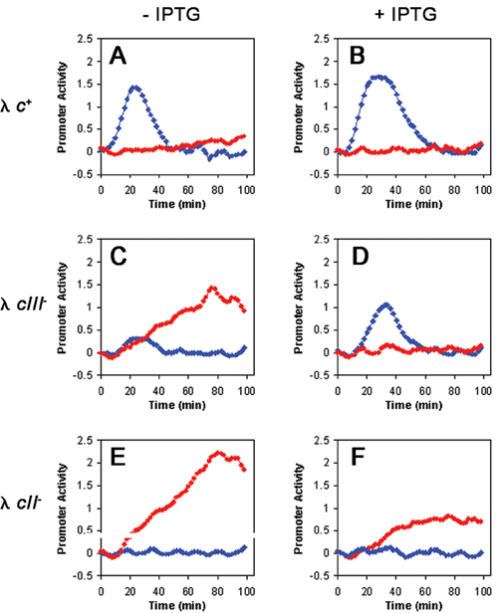
CIII levels effect on CII and Q activity *in vivo.* CII activity is reported by pE-*gfp* fusions (blue diamonds), whereas Q activity is reported by pR‘-tR‘-*gfp* fusions (red circles). Promoter activity is given as a function of time of infected cultures carrying the pCTCIII plasmid in the presence or absence of 0.1mM IPTG (B, D, F or A, C, E respectively) with λ*c+* (A, B), λ*cIII^−^* (C, D), and λ*cII^−^* (E, F). All measurements were carried out as described in the materials and methods at a MOI of 6.

Genetic experiments indicated that approximately 3-fold increase in the translation of CIII greatly favors the lysogenic response [29]. To determine how the continuous supply of CIII would affect CII activity we infected cells carrying a double plasmid system with λ strains. A compatible plasmid (pCTCIII) carrying the *cIII* gene under the *pTac* inducible promoter was introduced into wild type cells carrying one of the λ promoter-*gfp* fusion plasmids. Following infection of these cells in the presence or absence of the IPTG inducer, the CII and Q activities were monitored (Figure 6, blue and red lines respectively). The results show that ectopic supply of CIII increases both the time and the amplitude of CII activity expressed by the infecting phages. As expected, the effect of CIII supplied in trans on CII and Q activities is much more dramatic in infection with a λ *cIII^−^* mutant than infection by the wild-type phage. Interestingly, ectopic addition of CIII reduces the output signal from the pR′-tR′-GFP plasmid even in the absence of CII. It is possible that by inducing the heat-shock response CIII can further modulate Q antitermination activity [13]. Consistent with this hypothesis, we found that following infection either by λ *c+* or by λ *cII^−^* at 42°C, a significant decrease in Q activity was observed (data not shown).

**Figure 7:**
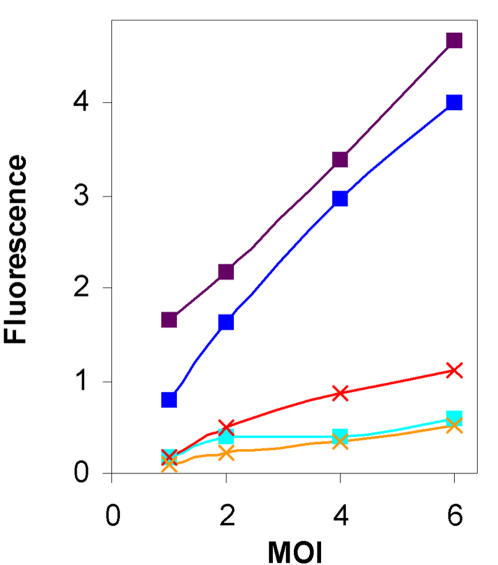
CIII levels affect multiplicity of infection. The total fluorescences obtain from the pE-GFP plasmid, 80 minutes after infection were plotted against the MOI of each experiment. λ*cIII^−^* infections of bacteria carrying the pCTCIII plasmid were followed in the absence (light blue squares) or presence of 0.1 mM IPTG (blue squares) or of 0.2 mM IPTG (purple squares). λ*c+* (red×symbol) and λ*cIII^−^* (orange×symbol) infections of bacteria without the pCTCIII plasmid are shown as reference.

The experimental setup allowed us to test the dependence of CII activity on the number of *cII* copies carried by the infecting phages in the presence of equal levels of CIII that is ectopically expressed. The results show a linear increase in CII activity as function of MOI (Figure 7). By comparing infection of λ c+ and λ *cIII^−^* phages, we found that the amount of CIII made from the infecting phage has an effect when MOI is greater than 4, and little or no effect at low MOI. However, CIII supplied from the plasmid increases CII function at all MOI's, allowing the phage to follow the lysogenic pathway even at low MOI. These results indicate that CIII can modulate the phage genetic network.

### CIII dominant negative mutant

We hypothesized that CIII oligomerization is required for efficient FtsH inhibition. We thus predict that it should be possible to identify CIII mutants that would be dominant negative due to formation of inactive CIII_mutant_-CIII_wild-type_ hetrooligomers. To find such a mutant we introduced, using recombineering [30], a set of five alanine substitutions into the essential region of CIII within the λ genome. The mutants S17A, R24A, L26A, R27A and R32A show a *cIII^−^* phenotype in plaque morphology and in CII activity, using the pE-GFP fluorescence assay (data not shown). Two mutants CIII_R27A_ and CIII_R32A_ were inserted into a plasmid under the tac promoter, for further analysis. Next, we confirmed by electrophoresis that these mutants formed oligomers at efficiency similar to that of wild type CIII (data not shown). We then tested their ability to cause dominant negative inhibition of wild type CIII activity. As shown in Table 1, the plating of wild type phage on a strain carrying the CIII_R32A_ plasmid leads to a clear plaque formation. Consistent with these results, we found reduction in the frequency of lysogeny from 10% to 0.22% in the strain carrying the R32A mutant plasmid. These results suggest that the CIII_R32A_ mutant inhibits wild type CIII activity and support the hypothesis that CIII oligomerization enhances FtsH inhibition.

**Table 1:** The dominant negative effect of CIII mutants as observed by plaque morphology.

Strain	λ c+	λ *cIII_67_*	λ *cII_68_*
AD16	T	C	C
AD16/pQECIII	T	T	C
AD16/pQECIII_R27A_	T	C	C
AD16/pQECIII_R32A_	C	C	C

AD16 strains carrying plasmids expressing wild type and mutated CIII via tac promoter were grown overnight at 32°C in LB or LB supplemented with 100 µg/ml ampicillin. A lawn of each strain was plated on TB plates in the presence of 10 µM IPTG and then spotted by different concentrations of the phages indicated in the table. C-clear plaques, T-turbid plaques.

### The host *sfhC21* mutation eliminates the bacteriostatic effect of CIII

LpxC catalyses the limiting reaction in lipopolysaccharide biosynthesis and controls the ratio between lipopolysaccharides and lipophospholipids in Gram-negative bacteria. Overproduction of LpxC causes toxic accumulation of membranes in the periplasm. LpxC levels are tightly regulated by the FtsH protease, which is therefore an essential protease in *E. coli.* [31]. Genetic experiments showed that *sfhC21*, a mutation that up-regulated the FabA dehydrase (of the type II fatty acid synthase system) suppresses a deletion in *ftsH* [31].

It was shown that overexpression of CIII impairs host cell growth and that this inhibition can be overcome by compensating high levels of FtsH capable of degrading CIII [13], [15], [32]. To identify if FtsH is the sole target responsible for CIII growth inhibition we constructed a plasmid carrying the pBAD-CIII-GFP fusion. This construct prevents colony formation when introduced into a wild type bacterial strain, indicating that the CIII-GFP fusion protein retains a strong inhibitory effect on cell growth (Table 2). However expression of CIII-GFP did not impair bacterial growth of the Δ*ftsH sfhC21* double mutant strain or the *sfhC21* single mutant. These findings corroborate the results shown by Ogura et al. [31] which demonstrated that the primary target of CIII in inhibiting cellular growth is FtsH proteolytic activity.

**Table 2:** CIII-GFP plasmid inhibits bacterial growth.

Strain	Growth ability	Growth ability carrying the CIII-GFP plasmid
Wild Type	+	-
Δ*ftsH*	-	NA
Δ*ftsH sfhC21*	+	+
*sfhC21*	+	+

The ability to grow on LB plates was tested in a set of isogenic strains of W3110 (Wild Type strain). To these strain the CIII-GFP plasmid was inserted by electroporation and the ability to form ampicillin resistant colonies was tested either at 30°C or 37°C by plating on LB plates with 100 µg/ml ampicillin in the absence of arabinose. The plus sign represent growth whereas the minus sign represents failure to grow. NA–not applicable.

## Discussion

The phage λ decision-making process has been studied extensively as a paradigm for genetic networks. The λ CIII inhibitor peptide is required for efficient lysogenic activation following phage infection. In this work we describe biochemically and genetically properties of this FtsH inhibitor. We identified similarities between CIII and the Bacillus SpoVM FtsH inhibitory peptide. Both inhibitors are degraded by FtsH and are found in association with the cell membrane [10], [11]. We found that CIII, as SpoVM, forms an amphipathic α helix. Sequence similarities suggest that the relatively conserved amphipathic α helix may be important for the inhibitory activity of both peptides.

It is interesting to note that CIII homologs are found in a growing number of temperate phages. As FtsH is highly conserved in prokaryotic organisms as well as in the mitochondria and the chloroplasts of eukaryotic cells, one might expect that the inhibitory function of this protease will also be conserved. However, no CIII-like proteins are found to be present in the genome database. It is possible that CIII-like functions having different primary sequences do exist or less likely, efficient temporal inhibition of FtsH did not find its use in bacterial evolution.

**Figure 8:**
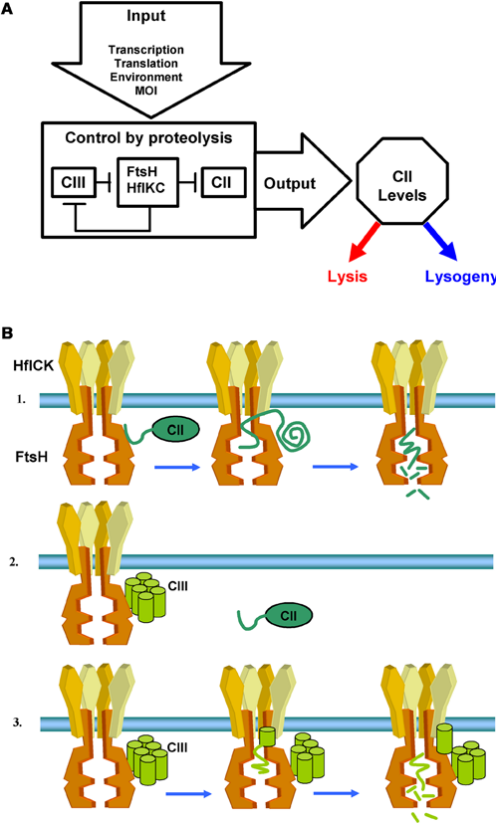
Schematic model for CIII activity. (A) The role of the CIII/CII/FtsH/HflKC module in the lambda genetic decision. The FtsH and HflKC complex regulates CII and CIII levels and CIII controls FtsH/HflKC proteolytic activity. (B) A schematic drawing of the CIII mechanism of inhibition (based on the model presented in [16]). In panel 1 in the absence of CIII, CII binds the FtsH protease and is translocated into the enzymatic cavity where it is first unfolded and then degraded. In panel 2, CIII binds the FtsH protease and prevents CII from binding. In panel 3, the CIII protein, which is found as an oligomer, is also subjected to degradation.

Both CII and CIII are tightly regulated at the levels of transcription and translation [19]. By inhibiting FtsH, CIII leads to an increase in the levels of CII activity. Thus, the CII/CIII/FtsH/HflKC act as a post-translational regulatory module (Figure 8A). Here we found that CIII acts as a competitive inhibitor of the host FtsH interfering with the binding of the CII substrate to the enzyme.

A number of biochemical properties of the CII/CIII/FtsH/HflKC module (Figure 8B) provide for its ability to finely tune CII levels, thus to tightly control the lysis-lysogeny decision. First, the FtsH/HflKC is present in the cell as huge, membrane-bound, highly active enzyme complexes, FtsH_6_HflKC_6_ of which there are probably less the 100 molecules in a cell [16]. Furthermore, FtsH degrades CII very rapidly without requiring adaptor or chaperone functions [33]. The CIII inhibitor is also subject to proteolysis by FtsH, which limits its activity to a short time window and allows for its rapid elimination once the lysogenic state is established. The elimination of CIII is required as CIII is deleterious to bacterial host. Our results suggest that FtsH is the only target of CIII responsible for the bacteriostatic effect of CIII.

The structure-function relationships of CIII are not known. The role of the amphipathic region may be for improved binding to FtsH or for the interaction with the cytoplasmic membrane favoring its binding to the membrane-bound FtsH [11]. We identified the ability of CIII to form oligomers, which may interact via the predicted coiled coil motif of this amphipathic region. The dominant negative effect of the CIII_R32A_ mutant over the wild type CIII strongly suggests that CIII functions *in vivo* in oligomeric form.

Many proteins of bacteriophage λ are regulated by rapid proteolysis by various proteases [5]. Interestingly, the key elements of the lysis lysogeny decision, the CII and CIII proteins, are mainly degraded by FtsH. We suggest that coevolutionary forces maintaining the balance between bacteria and the infecting phages preferred cells that carry the active protease critical for the regulation of lysis-lysogeny decision. The fittest mechanism was obtained by selecting the only essential ATP-dependent protease in *E. coli*.

## Materials and methods

### Bacterial strains and plasmids

The *ftsH* deletion strain is W3110 *sfhC* ZAD220::Tn10 *ftsH*3::*Kan*R [31]. The *hflK* mutant strain is W3110 *hflK*::*Kan*R prepared by P1 transduction. AD16 is strain carrying *lacI^q^* [34] The expression systems and purification processes of the FtsH, CII and CIII proteins were described before [12]. The CIII-GFP plasmid was constructed by insertion of the CIII gene into the pJHK5 plasmid (received from Marcia Goldberg) using the EcoRI and PstI restrictions sites for the 5′ and 3′ respectively. The lambda promoters-GFP fusion plasmids [28] were used for monitoring the lambda infection process. Plasmid pCTCIII [35] carries the λ cIII gene downstream of the p*Tac* promoter such that expression of the λ cIII gene can be conditionally induced by addition of the isopropyl-thio-β-D-galactopyranoside (IPTG) inducer. The point mutations in the *cIII* gene were introduced using single strand DNA oligos as described [30] and selected by formation of clear plaques. The mutations were confirmed by sequencing. The *cIII* mutant genes were amplified by PCR and cloned into the pQE30 plasmid (Qiagen) as described for the wild type CIII [12].

### Fluorescence assays

Strains carrying both one of the promoter-gfp plasmids and the pCT-CIII plasmid were grown overnight in LB medium supplemented with 0.2% maltose, 50 µg/ml ampicillin and 20 µg/ml chloramphenicol. Cells were concentrated by centrifugation to 6×10^9^ cells per ml, and 25 µl samples then were infected with different amounts of freshly prepared phage lysates (at the specified MOI) on ice. After incubation for 30 min on ice, the infected cells were diluted to 0.5 ml in M9 supplemented with 0.5% glycerol, 0.2% maltose, 0.01% B1, 50 µg/ml ampicillin and 20 µg/ml chloramphenicol, in the presence or absence of isopropyl -D-thiogalactoside (IPTG) as specified. Immediately after dilution, 200 µl cultures (6×10^7^ cells) were assayed in duplicates in 96-well plates for GFP activity. Plates were incubated at 37°C and assayed at 2-min intervals in a SPECTRAFluor Plus fluorimeter (Tecan, Maennedorf, Switzerland) with a short shaking interval between assays. The FtsH and HflK mutants experiments were done similarly with the exception that no chloramphenicol or IPTG were used in these experiments. The data analysis was carried out as described based on the average of two experiments [28].

### Circular Dichroism

The CD spectra (wavelengths 195–255nm) of a highly purified preparation of the His_6_-CIII diluted in PBS solution were recorded in a 0.5 mm path-length cuvette at 37°C on a Jobin et Yvon CD 6 spectropolarimeter with sampling intervals of 1 nm. The average of six scans is shown after smoothing the data by averaging each wavelength point with the previous two and next two points.

### *In vitro* assays

Proteolysis assays of GST-FtsH were performed as described [33]. Cross-linking reactions contained 4 µM CII, 1 µM GST-FtsH, CIII in different concentrations and 0.1mg/ml BSA in PBS solution. The reactions were incubated in the presence or absence of 10mM AMP-PNP (no nucleotide dependence was observed) for 5 minutes at 42°C. 0.25% Glutaraldehyde was added and the reaction was incubated for two more minutes at 42°C and terminated by the addition of glycine to a final concentration of 200 mM and transferring the reaction mix to 0°C. For CIII oligomerization 10 µM of His_6_-CIII were incubated in PBS solution with 0.1 mg/ml BSA at 42°C. The cross-linking reaction started by addition of 0.25% Glutaraldehyde and was stopped by 200 mM Glycine and transferring the reaction mix to 0°C.

### Frequency of lysogeny assay

Bacterial cultures (AD16; AD16/pCIII_R32A_) were grown, in LB medium and in LB-ampicilin medium, respectively. λ *c+ kn^R^* phage [12] was adsorbed on ice for 30 min, at a multiplicity of 0.01. The infected cells were diluted into LB medium in the presence of 10 µM IPTG isopropyl β-d-thiogalactoside and incubated at 37°C for 45 min before spreading on LB plates containing 30 µg/ml kanamycin. Lysogens were scored as percentage Km^R^ colonies of the input-infecting phage.
